# Device- and Nondevice-Guided Slow Breathing to Reduce Blood Pressure in Patients with Hypertension: Protocol for a Systematic Review and Meta-analysis

**DOI:** 10.2196/33579

**Published:** 2022-03-04

**Authors:** Kamila Shelry de Freitas Gonçalves, Ana Carolina Queiroz Godoy Daniel, José Luiz Tatagiba Lamas, Henrique Ceretta Oliveira, Renata C C P Silveira, Lyne Cloutier, Eugenia Velludo Veiga

**Affiliations:** 1 WHO Collaborating Centre for Nursing Research Development University of São Paulo Ribeirão Preto Brazil; 2 School of Nursing University of Campinas Campinas Brazil; 3 Département des sciences infirmières Université du Québec à Trois-Rivières Quebec, QC Canada

**Keywords:** hypertension, breathing exercises, device-guided breathing, respirate, systematic review, physical therapy, blood pressure, clinical decision making, health care professional, physiotherapy

## Abstract

**Background:**

Physiotherapy can include both device-guided slow breathing (DGSB) and nondevice-guided slow breathing (NDGSB) in the treatment of systemic arterial hypertension.

**Objective:**

The aim of this study is to summarize the effects of DGSB on blood pressure levels of patients with hypertension based on the published literature to date.

**Methods:**

A systematic search of all published randomized controlled trials (RCTs) on the effects of device-guided and nondevice-guided slow breathing in patients with hypertension, without language restriction, was carried out up to a publication date of January 2020 in nine databases: PubMed/MEDLINE, Latin American and Caribbean Health Sciences Literature (LILACS), EMBASE, CENTRAL (Cochrane Central Register of Controlled Trials), Physiotherapy Evidence Database (PEDro), CINAHL (Cumulative Index to Nursing and Allied Health Literature), Scopus, Web of Science, and Livivo. Clinical trial records databases (ClinicalTrials.gov), and bases for the open gray literature, including Gray Literature Report and ProQuest Central (Citation, Abstract or Indexing, and Dissertations and Theses), were also searched for potentially eligible RCTs. The quality assessment of the included studies will be performed using the Cochrane Risk of Bias Tool for Randomized Trials. The overall quality of the evidence for each outcome will be assessed using the GRADE (Grading of Recommendations, Development and Evaluation) system.

**Results:**

As of December 2021, the review was completed and all data from continuous variables referring to blood pressure values (mmHg) were synthesized.

**Conclusions:**

This systematic review will provide a summary of the current evidence on the effects of both DGSB and NDGSB on blood pressure levels. This information can contribute to decision-making by health professionals related to the use of these interventions in patients with hypertension.

**Trial Registration:**

PROSPERO (Prospective International Register of Systematic Reviews) CRD42020147554; https://www.crd.york.ac.uk/prospero/display_record.php?RecordID=147554

**International Registered Report Identifier (IRRID):**

RR1-10.2196/33579

## Introduction

Hypertension is a multifactorial chronic disease, and is the main risk factor for the development of cardiovascular diseases and chronic kidney disease. Hypertension affects 32% of adults and more than 60% of the elderly, being responsible for half of the deaths from cardiovascular disease (CVD) in Brazil [1]. In addition, the complications of hypertension can lead to decreased work productivity and family income [1,2]. In high-income countries such as Canada, the hypertension prevalence has declined; in middle-income countries such as those of Latin America, Asia, the Middle East, and North Africa, detection and treatment hypertension have enhanced, whereas low detection and treatment rates persist in the poorest nations such as those of sub-Saharan Africa and Oceania [1,3,4].

Considering the high prevalence rates, the treatment of hypertension is necessary, not only to reduce blood pressure levels but also to prevent the development of CVD, cerebrovascular diseases, and kidney diseases. This treatment can be medication, which will be determined according to the blood pressure values obtained either in medical consultations or at home; cardiovascular risk factors; and the presence of target organ damage identified during anamnesis. Nonpharmacological treatment has also been shown to be effective in reducing blood pressure levels in patients with hypertension [1-4], including body weight control, the establishment of healthy eating habits (specifically reducing salt consumption), alcohol consumption control, smoking cessation, stress control, aerobic and isometric physical exercises, and slow breathing with or without device guide [1-4].

The physiotherapy prescription for the treatment of hypertension may include both exercise and device-guided slow breathing (DGSB) or nondevice-guided slow breathing (NDGSB). These breathing exercises consist of slow and deep breathing with 6 to 10 breaths per minute, and can be performed with or without a guiding device. Isometric exercises have been shown to be effective in reducing blood pressure levels, along with aerobic and dynamic exercises [4-6]. However, the application of DGSB remains controversial. Since DGSB activates cardiac and pulmonary stretching receptors, decreases sympathetic activity, and increases parasympathetic activity and vagal tone, thus changing the heart rate and blood pressure, it would be clinically sound to consider that it would reduce blood pressure levels. With blood pressure reduction, there is an increase in baroreflex sensitivity, which promotes improvements in the autonomic balance of patients with hypertension [7].

The American Heart Association reported that there is no strong evidence on the effectiveness of DGSB, whereas the 8th Brazilian Hypertension Guidelines reported a IIa degree of recommendation, level of evidence A [1,4]. A review [8] indicated that there is currently insufficient evidence of data to recommend the routine use of DGSB in patients with hypertension, even though this device has been cleared by the US Food and Drug Administration and the UK National Health Service. In their review, Cernes et al [9] stated that DGSB, as long as it is monitored by a health professional, can be recommended for patients with hypertension who cannot obtain full control of their blood pressure with drug treatment or cannot tolerate the side effects of treatment. Barros et al [10] performed a controlled clinical study with 15 individuals in the control group and 17 in the experimental group, who practiced DGSB for 15 to 20 minutes a day, with 6 to 10 breaths per minute, and concluded that DGSB, in the long term, did not reduce blood pressure values, catecholamine levels, or muscle sympathetic nerve activity in patients with hypertension. However, the use of DGSB was indicated in the 7th Brazilian Hypertension Guidelines [11].

Recommendations for the use of DGSB or NDGSB in clinical practice should be guided by a systematic, high-quality literature review. Recently, Chaddha et al [12] published a review that fulfills this requirement, which compared DGSB to NDGSB (*pranayama*) for 4 weeks in patients with prehypertension and hypertension. The review included 17 studies, with systolic blood pressure reported in 1017 subjects and diastolic blood pressure reported in 964 subjects. Although interesting, this review did not specifically include patients with hypertension and exclusively compared DGSB to *pranayama*. Therefore, a systematic review of the antihypertensive effects of DGSB or NDGSB applied by physical therapists is necessary to provide the best evidence available to clinical physical therapists and patients with hypertension. In addition, it is also important to summarize the evidence on the effectiveness of DGSB or NDGSB compared to usual care.

Accordingly, the aim of this systematic review and meta-analysis is to summarize the effects of DGSB on blood pressure levels of patients with hypertension compared with control conditions (such as minimal intervention, usual care, placebo, and no treatment), other interventions (NDGSB), and when used as an adjunct to other treatments (medication). Thus, the research question for this systematic review of randomized controlled trials (RCTs) is: What are the effects of the prolonged use of device-guided or nondevice-guided slow breathing compared to usual care on the blood pressure values of patients with hypertension?

## Methods

### Study Design

The design of this systematic review is inspired by the recommendations of the Cochrane Handbook of Systematic Reviews [13] and the PRISMA (Preferred Reporting Items for Systematic Review and Meta-Analysis) guidelines [14].

The articles were selected based on inclusion criteria according to the type of study, participants, and intervention.

### Inclusion Criteria

#### Types of Studies

RCTs published up to January 2020 were included in this systematic review, without language or year of publication restrictions.

#### Types of Participants

Studies including patients with hypertension, with or without comorbidity, who were over 18 years old, of both sexes, and with or without antihypertensive medication treatment were included for review.

#### Types of Interventions

Interventions considered had to involve DGSB and NDGSB compared to control conditions (such as minimal intervention, usual care, placebo, and no treatment) and interventions as an adjunct to other treatments (medication). Any dosage of device-guided breathing treatment was accepted. Regarding the follow-up time, studies with a duration of a minimum of 8 weeks were considered.

### Exclusion Criteria

RCTs that also used other interventions along with DGSB/NDGSB, such as physical activity (aerobic exercises, Tai Chi, resistance training, and isometric exercises); salt reduction and salt substitution; stress control (meditation, Qigong, yoga, progressive muscle and reduction programs for attention-based stress disorders); dietary interventions, including a dietary approach to stop hypertension, low-carbohydrate diet, Mediterranean diet, high-protein diet, low-fat diet, vegetarian diet, paleolithic diet, and low index glycemic/load; and lifestyle interventions (comprehensive lifestyle modification, smoking cessation, alcohol restriction, sleep, home heating, and weight loss) were excluded since it was not possible to identify the specific effect of DGSB/NDGSB in such studies.

### Outcome Measures

The primary outcome was the systolic blood pressure and diastolic blood pressure values (measured at home, in the office, or by ambulatory blood pressure monitoring), expressed in mmHg, measured after the interventions, as well as their variations. The secondary outcome was a reduction in the quantity/dosage of drugs administered to control hypertension, if relevant.

### Identification and Selection of Studies

A systematic search of all published RCTs on the effects of device and nondevice-guided slow breathing in patients with hypertension, without language restriction, was carried out until January 2020 in nine databases: Pubmed/MEDLINE, Latin American and Caribbean Health Sciences Literature (LILACS), EMBASE, CENTRAL (Cochrane Central Register of Controlled Trials), Physiotherapy Evidence Database (PEDro), CINAHL (Cumulative Index to Nursing and Allied Health Literature), Scopus, Web of Science, and Livivo. Clinical trial record databases (ClinicalTrials.gov), and sites for the open grey literature, such as Gray Literature Report and ProQuest Central (Citation, Abstract or Indexing, and Dissertations and Theses), were also searched. Completed and ongoing RCTs were searched up to January 2020 and, when possible, only peer-review papers were included, because the grey literature was also searched.

### Search Strategy

An example of the search strategy used in PubMed/MEDLINE is shown in Textbox 1.

Two reviewers independently analyzed all titles and abstracts retrieved with the search. When there was agreement on a particular record, the full text was analyzed by both reviewers, according to the eligibility criteria. In the presence of disagreement between the reviewers, a third reviewer was convened. When additional information was needed, the authors of the potentially eligible studies were contacted.

**Search strategy in the PubMed/MEDLINE database.**

Hypertension [Mesh: NoExp], hypertensi*, high blood pressure, high blood pressures, blood pressure [Mesh], blood pressure, arterial pressures, arterial tension, arterial tensions, Arterial Pressure [Mesh], Arterial pressure, arterial blood pressure, arterial blood pressures, elevated blood pressure, acute hypertension, arterial hypertension, cardiovascular hypertension, controlled hypertension, hypertensive disease, systemic hypertension, increased blood pressure, artery blood pressure, artery pressure, systemic arterial pressure, systemic artery pressure, Hypertension [Mesh: NoExp] OR hypertensi* OR high blood pressure OR high blood pressures OR blood pressure [Mesh] OR blood pressure OR arterial pressures OR arterial tension OR arterial tensions OR Arterial Pressure [Mesh] OR Arterial pressure OR arterial blood pressure OR arterial blood pressures OR elevated blood pressure OR acute hypertension OR arterial hypertension OR cardiovascular hypertension OR controlled hypertension OR hypertensive disease OR systemic hypertension OR increased blood pressure OR artery blood pressure OR artery pressure OR systemic arterial pressure OR systemic artery pressure, Breathing Exercises [Mesh: NoExp], breathing exercises, respiratory muscle training, device-guided breathing, loaded breathing, slow breathing exercises, paced breathing, controlled breathing, breathing exercise, breathing therapy, chest physical therapy, chest physiotherapy, respiration exercise, respiration therapy, respiratory exercise, respiratory physiotherapy, deep inspiration, deep respiration, deep breathing, Breathing Exercises [Mesh: NoExp] OR breathing exercises OR respiratory muscle training OR device-guided breathing OR loaded breathing OR slow breathing exercises OR paced breathing OR controlled breathing OR breathing exercise OR breathing therapy OR chest physical therapy OR chest physiotherapy OR respiration exercise OR respiration therapy OR respiratory exercise OR respiratory physiotherapy OR deep inspiration OR deep respiration OR deep breathing OR deep respiration OR deep breathing, Hypertension [Mesh: NoExp] OR hypertensi* OR high blood pressure OR high blood pressures OR blood pressure [Mesh] OR blood pressure OR arterial pressures OR arterial tension OR arterial tensions OR Arterial Pressure [Mesh] OR Arterial pressure OR arterial blood pressure OR arterial blood pressures OR elevated blood pressure OR acute hypertension OR arterial hypertension OR cardiovascular hypertension OR controlled hypertension OR hypertensive disease OR systemic hypertension OR increased blood pressure OR artery blood pressure OR artery pressure OR systemic arterial pressure OR systemic artery pressure AND Breathing Exercises [Mesh: NoExp] OR breathing exercises OR respiratory muscle training OR device-guided breathing OR loaded breathing OR slow breathing exercises OR paced breathing OR controlled breathing OR breathing exercise OR breathing therapy OR chest physical therapy OR chest physiotherapy OR respiration exercise OR respiration therapy OR respiratory exercise OR respiratory physiotherapy OR deep inspiration OR deep respiration OR deep breathing OR deep respiration OR deep breathing

Two reviewers independently extracted the following data from the included trials: author, publication date, country of publication, study type, sample size, participant characteristics (age, gender), use or not of antihypertensive medications, presence of comorbidities, categories of blood pressure, details of intervention (type of device used in the DGSB, whether DGSB was performed with or without load, how the NDGSB was performed, breaths per minute for DGSB and NDGSB, time of use of the device per day and for how many months), details for blood pressure measurement (device used, type of measurement [home or office], and protocol used for measurement, including preparation), and outcome measures (systolic and diastolic blood pressure). A third reviewer was called in case of disagreement. When necessary, the authors of RCTs included were contacted to provide additional information.

### Assessment of Study Characteristics

The quality assessment of the included studies was conducted using the Cochrane Risk of Bias Tool for Randomized Trials (RoB2) [15], which includes a randomization process, deviations from the intended interventions, conflicting result data, result measurement, selection of the reported result, and general biases. The same two reviewers performed an independent assessment. Disagreements between reviewers were resolved by discussion and, if necessary, the opinion of a third reviewer was requested. The same two reviewers performed data extraction, using standardized forms regarding the methodological characteristics of the studies, interventions, and results. Disagreements were again resolved by discussion and, if necessary, the opinion of a third reviewer was requested.

### Data Analysis

All data from continuous variables referring to blood pressure values (mmHg) will be synthesized according to the mean difference and respective 95% CIs. Standard deviations were also extracted from the studies for analysis.

The effects of interventions on blood pressure values will be analyzed separately. The data will be evaluated according to the type of intervention (DGSB or NDGSB); however, only studies lasting at least 8 weeks will be considered for meta-analysis (results evaluated after 8 weeks of randomization). Results where there was an intention-to-treat analysis will be used whenever possible.

The presence of statistical heterogeneity between RCTs will be assessed using the *I^2^* statistic. The quality of the evidence will be considered inconsistent if considerable heterogeneity between the groups (*I^2^*>50%) is observed. When sufficient evidence is available, a funnel graph will be used to investigate possible publication bias**.**

### Data Synthesis

The overall quality of the evidence for each outcome will be assessed using the GRADE (Grading of Recommendations, Development and Evaluation) [16] system, regardless of whether or not the information is sufficient to summarize the data in a quantitative analysis. The following five factors will be considered when classifying the quality of the evidence: risk of bias (>25% of the RCTs included in the comparison are classified as having a high risk of bias), inconsistency (*I^2^*>50%), indirectness (>50% of participants were not related to the target audience trial), imprecision (<400 participants in the comparison for continuous outcomes), and publication bias (assessed using a funnel plot when >10 trials are in the same comparison). For each factor not met, the quality of the evidence is reduced by one level (from high to moderate, low, or very low). Single trial comparisons (<400 participants for continuous results) were found to be inconsistent and inaccurate, providing “low quality evidence,” which could be downgraded to “very low quality evidence” if limitations are identified in relation to the risk of bias [16].

The quality of the evidence will be categorized as follows: the evidence is of high quality if the results are consistent in ≥75% of the participants, with a low risk of bias, without publication bias, and with consistent direct and accurate data; further research is unlikely to alter the estimate or confidence in such results. The evidence will be considered to be of moderate quality when one of the five classification factors above is met; further research can alter the estimated effect and impact confidence in the effect in this case. The evidence will be considered to be of poor quality when two of the five classification factors are not met. In this situation, future research is likely to alter the estimated effect and have a significant impact on confidence in the effect. The evidence will be considered to be of very low quality when three of the five classification factors are not met, as any estimate of effect is uncertain in this case [16].

## Results

As of December 2021, the review is complete and all data from continuous variables referring to blood pressure values (in mmHg) have been synthesized.

## Discussion

This systematic review aims to provide the best available evidence on the effectiveness of DGSB or NDGSB in patients with hypertension, as well as whether DGSB/NDGSB allows for a reduction in the amount/dosage of antihypertensive medication administration. All recommendations in the Cochrane Manual of Systematic Reviews will be followed to ensure that the review is of high quality. It is believed that the results of this systematic review will be important because both DGSB and NDGSB are accessible and can be performed even at home, as long as the use is prescribed and guided by a physical therapist.

In addition, this review is the first to assess NDGSB unrelated to the effect of *pranayama* on the blood pressure of patients with hypertension, since this type of intervention is routinely used in a physiotherapist’s clinical practice, following a completely different method from that adopted in yoga. Therefore, this evidence will inform health care professionals and patients about the potential benefits of this intervention. This review may also identify gaps in the literature that can be addressed in future studies.

## Abbreviations

CENTRAL: Cochrane Central Register of Controlled Trials
CINAHL: Cumulative Index to Nursing and Allied Health Literature
CVD: cardiovascular disease
DGSB: device-guided slow breathing
GRADE: Grading of Recommendations, Development and Evaluation
LILACS: Literatura Latino-Americana e do Caribe em Ciências da Saúde
NDGSB: nondevice-guided slow breathing
PEDro: Physiotherapy Evidence Database
PRISMA: Preferred Reporting Items for Systematic Review and Meta-Analysis
RCT: randomized controlled trial
RoB2: Cochrane Risk of Bias Tool for Randomized Trials
